# Detection of asymptomatic celiac disease in two siblings from a mother with non-celiac gluten sensitivity 

**Published:** 2018

**Authors:** Umberto Volta, Giacomo Caio, Roberto Manfredini, Roberto De Giorgio

**Affiliations:** 1 *Department of Medical and Surgical Sciences, University of Bologna, Italy*; 2 *Mucosal Immunology and Biology Research Center, Massachusetts General Hospital, Harvard Medical School, Boston, MA, USA*; 3 *Department of Medical Sciences, Clinica Medica Unit, University of Ferrara, Italy *

**Keywords:** Celiac disease, Non-celiac gluten sensitivity, Screening, Serology, Relatives, Familiarity

## Abstract

Non-celiac gluten sensitivity and celiac disease are known to be two distinct clinical entities, however, non-celiac gluten sensitivity has been detected in a proportion of first-degree relatives of celiac patients. Herein for the first time we describe the occurrence of asymptomatic celiac disease in two siblings, a girl and a boy, whose mother suffered from a proven non-celiac gluten sensitivity. Both the 12-year old girl and 9-year old boy were positive for anti-endomysial and anti-tissue transglutaminase antibodies of IgA class at a very high and low titer, respectively. Duodenal biopsy confirmed the diagnosis of active celiac disease (severe villous flattening) in the girl, whereas her brother had Marsh 1 lesion consistent with a potential celiac disease. This case report indicates that antibody screening for celiac disease can be recommended in any symptomatic or asymptomatic first-degree relatives of patients with non-celiac gluten sensitivity.

## Introduction

 The spectrum of gluten-related disorders has acquired a new clinical condition defined as non-celiac gluten sensitivity (NCGS) (1). In sensitized patients gluten ingestion evokes intestinal and extra-intestinal symptoms in a time frame of hours or days. Typically patients with NCGS diagnosis test negative for celiac disease (CD) serology, i.e. anti-tissue transglutaminase (tTGA) and anti-endomysial antibodies (EmA) as well as for villous flattening at duodenal biopsy histopathology (2). NCGS has been described as having different pathogenetic mechanisms from CD, the first condition being characterized by a prominent innate immunity response, whilst the second showing a dominant role of adaptive immunity. For such reasons current nosology considers these two diseases as distinct clinical entities (3). Nonetheless, a systematic review of the literature showed that 136 (15.7%) over 867 patients with NCGS, identified in four studies, had a family history of CD (4). The vast majority of them were first-degree relatives of CD patients and the diagnosis of NCGS followed that of CD in at least one family member (5-8). To our knowledge, there is no evidence of CD detection in family members with proven NCGS. Herein, we report the very peculiar finding of an unexpected CD in two asymptomatic siblings following NCGS diagnosis in their mother. 

## Case Report

A 43-year old woman was referred to our outpatient clinic due to gastrointestinal symptoms (i.e. abdominal pain and bloating, diarrhea, gastro-esophageal reflux) and extra-intestinal manifestations (weakness, headache, foggy mind and limb numbness, skin rash, fibromyalgia-like symptoms and anemia) triggered by gluten and wheat ingestion. Symptoms started three years before, when the patient was 40 years old. IgA tTGA and EMA tested negative as well as duodenal biopsy showed a normal mucosal architecture on a gluten containing diet, thus ruling out CD diagnosis. Wheat allergy was excluded by means of IgE to gluten and wheat as well as by skin prick tests. As part of a thorough diagnostic work-up, the patient was found to be positive for HLA-DR7 and -DQ2 haplotype. Other laboratory data revealed positivity for antibodies to native gliadin of IgG class (AGA IgG, twice the upper normal limit; conversely, deamidated gliadin peptide IgG antibodies were negative) and low levels of folic acid, ferritin and vitamin D. Thyroid function tests disclosed a condition of autoimmune thyroiditis without hypothyroidism. An open 6-week trial with gluten-free diet (GFD) led to a significant symptomatic improvement in a few days and the patient remained symptom-free on GFD. The diagnosis of NCGS was validated by means of a double-blind placebo-controlled cross-over trial as previously described (9). The patient was advised to follow a strict GFD which led to a significant improvement of her clinical picture along with disappearance of IgG AGA. Notably, following GFD a significant improvement of folic acid, ferritin and vitamin D levels was observed at 6-month follow-up.

Concerning the family history, the patient had two children, a 12-year old daughter and a 9-year-old son. None of them complained of gastrointestinal and extra-intestinal symptoms and they showed a normal growth without signs of short stature and weight loss. Laboratory data of these two children were unremarkable with normal value of hemoglobin, red blood cells, white cells and platelets. Values of vitamin D3, ferritin and folic acid were in the normal range in both children. 

**Figure 1:**
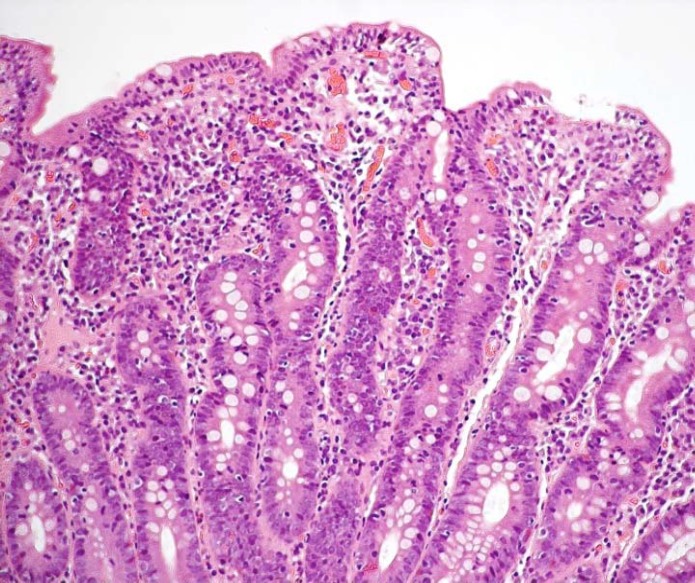
Duodenal mucosa with grade 3c subtotal villous atrophy (according to Marsh-Oberhüber classification), increased number of intraepithelial lymphocytes (>25 IELs/100 epithelial cells) and crypt hypertrophy in the 12-year old girl with a gluten sensitive mother (H&E staining; magnification 40x

CD antibody screening turned to be positive in both children, despite they were asymptomatic and with normal laboratory data. The 12-year old girl showed positivity for tTGA of IgA class at a very high titer (>10 times the upper normal limit) associated with EMA of IgA class. The genetic haplotype of this girl was positive for HLA-DR3 and -DQ2, corroborating the diagnosis of CD. Duodenal biopsy confirmed an active CD by showing a subtotal villous flattening (Marsh III) (10, 11) (Figure 1). The 9-year old boy was positive at a low titer for tTGA of IgA class (1.5 times the upper normal limit) associated with a weak IgA EMA positivity. 

**Figure 2:**
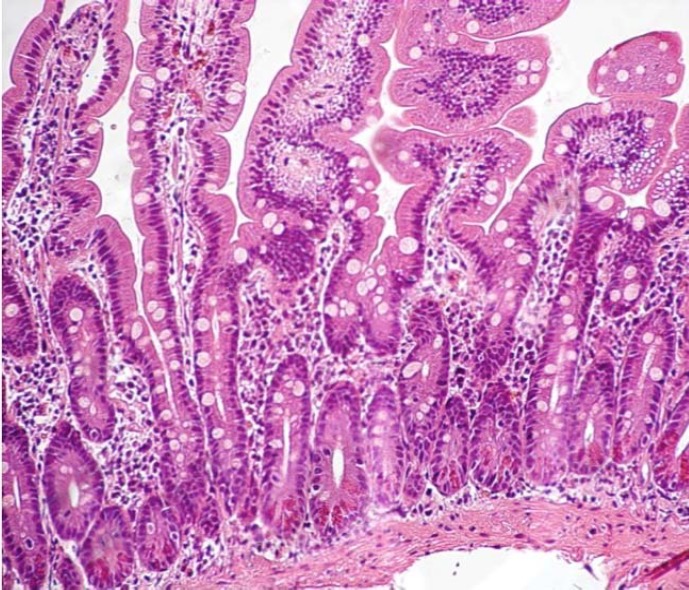
Duodenal mucosa with grade 1 infiltrative lesion (according to Marsh-Oberhüber classification), increased number of intraepithelial lymphocytes (>25 IELs/100 epithelial cells), normal villi and crypts in the 9-year old boy with a non-celiac gluten sensitive mother (H&E staining; magnification 40x

Genetic testing highlighted the same genetic pattern of the sister. Duodenal biopsy revealed the presence of normal villi, with a villous/crypt ratio >3:1, but with an increased number of intraepithelial lymphocytes (IELs) (55 IELs/100 epithelial cells), consistent with a diagnosis of potential CD (Figure 2). Both siblings were also positive for antinuclear antibodies (ANA) detected by indirect immunofluorescence on HEp2 cells. In the boy, ANA tests showed a speckled pattern at a low titer (1:80) with negativity for anti-extractable nuclear antigen antibodies (ENA). The girl showed ANA positivity with homogeneous pattern at a very high titer (1:640) associated with ENA positivity (SSA-Ro52 +++). Both children underwent a rheumatological evaluation which was unremarkable for connective tissue disease. Due to the condition of active CD with the typical villous flattening, the girl started a GFD, which led to a significant decrease of antibody titer after 6 months. In contrast, the boy was left on a gluten containing diet as suggested for asymptomatic potential CD (12). Patients involved in this case report signed informed consent to grant permission of the inclusion of their data in the study in an anonymous form. 

## Discussion

CD and NCGS are two gluten-related disorders which differ each other for pathogenic mechanisms, small bowel histopathology and serological markers, although the clinical picture can be impressively overlapping (1, 2, 13). Indeed, gastrointestinal symptoms such as gastroesophageal reflux, abdominal pain and bloating, diarrhea and constipation (or alternating bowel habit) and extra-intestinal manifestations, such as iron-deficiency anemia, osteoporosis, aphthous stomatitis, arthromyalgias, headache, foggy mind, dermatitis, are found in both conditions (7). Established cases of NCGS can be commonly diagnosed in families with one or more CD subjects (5-8). Indeed, first-degree relatives of CD patients represent a group at high risk for NCGS since a significant proportion of them complain of gastrointestinal, neurological, dermatological and fibromyalgia-like symptoms induced by gluten ingestion in the absence of any diagnostic criteria matching CD. However, whether CD can be diagnosed in first-degree relatives of NCGS patients remain unsettled. Nowadays, CD onset is frequently asymptomatic and this can explain why many CD diagnoses are still missed (14). 

**Table 1:** Clinical features, celiac serology, duodenal biopsy, genetic testing and other autoantibodies in the two siblings identified as celiacs after the diagnosis of non-celiac gluten sensitivity in their mother.

Age	Gender	Symptoms	tTGA IgA (n.v. <10 AU)	EMA IgA	HLA	Duodenal biopsy*	Other autoantibodies	Diagnosis
12 years	female	absent	105 AU	++++	DR3-DQ2	grade 3c	ANA 1:640 homogeneous pattern; ENA SSA-Ro52 +++; Anti-DNA negative	celiac disease
9 years	male	absent	15 AU	+ / - (weak positivity)	DR3-DQ2	grade 1	ANA 1:80 speckled pattern; ENA negative; Anti-DNA negative	potential celiac disease

Abbreviations: AU, arbitrary units; tTGA: anti-tissue transglutaminase antibodies; EMA: anti-endomysial antibodies; n.v.: normal value; HLA: human leukocyte antigens;

* duodenal biopsy classified according to Marsh classification; ANA: antinuclear antibodies on HEp2 cells; ENA: antibodies to extractable nuclear antigens.

This case report focuses on the finding of asymptomatic CD in two siblings of a mother with NCGS (Table 1). The diagnosis of NCGS was confirmed by a double blind placebo-controlled cross over challenge with gluten. The evidence that a subset of CD patients may be completely asymptomatic (hence ‘silent CD’) along with that NCGS is relatively common in first-degree relatives of CD patients (4), led us to test the two children for CD serology, being born from a mother suffering from NCGS. We thought this approach worthy on the basis of a conceptual transient property which applies as follows: as CD patients may have relatives with NCGS, likewise NCGS patients may have CD patients in their first degree relatives. Notably, the positive celiac serology allowed to establish CD diagnosis in the two asymptomatic children, one (the 12-year old girl) with a high tTGA titer which was correlated with a severe villous flattening, whereas the other (the 9-year old boy) with a low tTGA titer indicated a mild mucosal inflammation supporting a potential CD diagnosis. In this line, both children showed a genetic predisposition to CD as reflected by HLA-DQ2 positivity. According to current standard of treatment, GFD was mandatory in the girl, while it was questionable in the boy who was left on a gluten containing diet (12, 14). A further good reason for starting GFD in the girl was because of her autoantibody profile characterized by the positivity for ANA/ENA since gluten restriction may halt the risk of autoimmune disorder development (15). While the girl’s follow up was a classic one as for any other CD patient, the boy was periodically checked up every six months by tTGA and EMA serology and a new duodenal biopsy is planned if symptoms occur or antibody titer arises (12).

As ancillary data, serological screening in the first-degree relatives of NCGS patients allowed us to identify CD in other two mothers with their daughters affected by NCGS. These two women, aged 55 and 63 years at the time of CD diagnosis, were both positive for tTGA IgA and EMA and showed a duodenal biopsy consistent with partial and subtotal villous flattening, respectively. One of them was completely asymptomatic, whereas the other one, with a long history of iron-deficiency anemia, had never been investigated.

In conclusion, the finding of CD in these two asymptomatic siblings with a mother suffering from NCGS highlights the possibility of CD in the first degree relatives of NCGS patients. Therefore, serological screening for CD in first-degree relatives of patients with NCGS might be indicated. Our cases illustrate the delicate interplay between NCGS and CD, which may open novel horizons in terms of clinical and pathophysiological perspectives. Further studies are eagerly awaited to establish the individual burden of each condition, i.e. whether CD may predispose to NCGS, or vice versa, in first-degree relatives.

## Conflict of interests

The authors declare that they have no conflict of interest.
